# Unraveling the Pathophysiology of Obesity-Related Insulin Resistance—A Perspective on “Adipose Tissue Inflammation Is Directly Linked to Obesity-Induced Insulin Resistance, while Gut Dysbiosis and Mitochondrial Dysfunction Are Not Required”

**DOI:** 10.1093/function/zqaa021

**Published:** 2020-10-05

**Authors:** Gijs H Goossens, Ellen E Blaak

**Affiliations:** Department of Human Biology, NUTRIM School of Nutrition and Translational Research in Metabolism, Maastricht University Medical Centre, Maastricht, The Netherlands

Obesity is a major determinant of many noncommunicable diseases (NCDs), including cardiovascular diseases, nonalcoholic fatty liver disease, Type 2 diabetes, and several types of cancer.^1^ A prolonged positive energy balance leading to weight gain and excess fat mass is frequently accompanied by adipose tissue dysfunction. It is well established that adipose tissue dysfunction, which is characterized by adipocyte hypertrophy, mitochondrial dysfunction, a proinflammatory phenotype, and insulin resistance, contributes to systemic low-grade inflammation, dyslipidemia, and excessive storage of lipids in skeletal muscle, the liver, pancreas, and the heart.^2–4^ More recently, studies in rodents and humans have provided evidence that microbes residing in the intestine may influence host metabolism via multiple mechanisms, among which the production of gut microbial metabolites, and it has been suggested that the gut microbiome might be a causative factor in obesity-related NCDs.^5^ Together, these pathophysiological processes have detrimental effects on whole-body glucose homeostasis and cardiometabolic disease risk.

Although adipose tissue dysfunction seems to play a critical role in the development and progression of low-grade systemic inflammation and cardiometabolic complications, it often coexists with perturbations in other organs involved in the regulation of inflammatory and/or metabolic status.^2^ This implies that it is often difficult to draw firm conclusions about causal involvement of tissue-specific impairments in the initiation of obesity-related insulin resistance. Findings from cross-sectional studies do not allow conclusions about causality, while longitudinal studies comparing high fat diet (HFD)-fed or genetically-induced obese rodents with lean (wild type) controls do not provide sufficient insight into the relative importance of tissue-specific metabolic and inflammatory perturbations, and often do not elucidate the sequelae of events in the pathophysiology of insulin resistance and impaired glucose homeostasis. The reason for this is that these rodent models of obesity very rapidly develop whole-body insulin resistance and glucose intolerance, the very high amount of lipids in the commonly used HFD can directly impact the gut microbiota composition, adipose tissue inflammation, and insulin signaling in peripheral tissues, and genetic deletion of certain proteins to induce obesity may also impact immunity. A better understanding of the upstream cellular mechanisms involved in the pathophysiology of obesity-related insulin resistance and glucose intolerance may pave the way for novel prevention and treatment strategies to combat obesity-related insulin resistance and NCDs.

In this issue of *Function*, Petrick et al.^6^ explored the importance of adipose inflammation, skeletal muscle mitochondrial dysfunction, and gut dysbiosis for obesity-induced insulin resistance and glucose intolerance, and aimed to dissect out the independent effect of obesity from lipid overload. To address this objective, they first characterized C57Bl/6J mice that spontaneously and rapidly develop obesity on a standard chow diet. This animal model of obesity appeared independent of leptin deficiency or mutations within the leptin receptor, and therefore does not replicate *ob/ob* or *db/db* mice.^7^ Rather, the C57Bl/6J mice spontaneously developed a polygenic form of obesity. Although the authors were not able to demonstrate which gene instigated obesity, these animals represent a very interesting model to identify the mechanisms underlying obesity-related insulin resistance and impaired glucose tolerance in the absence of aberrant leptin signaling or HDF-feeding.

The team of investigators next assessed the changes in skeletal muscle, adipose tissue, liver, the intestinal microbiome, whole-body substrate oxidation, and glucose homeostasis in these spontaneously obese mice relative to lean controls on different diets. The spontaneously obese C57Bl/6J mice were characterized by hyperphagia, adipocyte hypertrophy, adipose inflammation, and mitochondrial redox imbalance, hepatic steatosis, and severe insulin resistance in adipose tissue, the liver, and skeletal muscle, despite consuming a standard rodent diet. Interestingly, however, the spontaneously obese mice showed normolipidemia, no signs of mitochondrial dysfunction in adipose tissue, skeletal muscle, the liver, and heart, and no differences in the gut microbiome relative to lean littermates. Interestingly, caloric restriction induced by *pair feeding* of a standard rodent diet in 5-week-old obese mice to lean controls did not alter white adipocyte size, macrophage/dendritic markers, or inflammatory markers. However, *pair feeding* markedly decreased the number of crown-like structures (reflecting macrophage number) and partially reduced markers of cytotoxic, helper and memory T cells, which was accompanied by improved insulin sensitivity and whole-body glucose tolerance. The authors go further with a crucial experiment to address the study objective and show intriguingly that feeding an obesogenic HFD to a separate subset of lean and obese mice not only elicited adipose tissue inflammation, but also altered the composition of the gut microbiome, induced skeletal muscle mitochondrial dysfunction, and evoked glucose intolerance compared to low fat diet feeding.

Altogether, the results from the elegant and well-performed experiments by Petrick et al.^6^ uncover a critical role for adipose tissue inflammation in obesity-related insulin resistance and glucose intolerance. In contrast, these data suggest that alterations in the gut microbiome and skeletal mitochondrial dysfunction seem dispensable for obesity-induced insulin resistance in spontaneously hyperphagic obese C57Bl/6J mice, and may rather be due to prolonged intake of an HFD.

A number of important issues raised by these series of experiments remain to be addressed. First, the spontaneously hyperphagic, obese C57Bl/6J mice not only developed adipose tissue inflammation but also hepatic steatosis. In addition, caloric restriction reduced liver lipid droplet size and mitigated some but not all proinflammatory markers in adipose tissue. Therefore, it can be questioned whether adipose tissue dysfunction is the culprit in obesity-related glucose intolerance, since lipid accumulation in the liver may also have significantly contributed to the metabolic phenotype observed in these animals, for example, through alterations in hepatokine secretion, or might even have driven adipose tissue dysfunction. Second, in contrast to the identical genetic background and similar laboratory conditions in the spontaneously hyperphagic, obese C57Bl/6J mice that were studied by Petrick et al.,^6^ the regulation of body weight and metabolic control in free-living humans is influenced by many internal and external factors. The impact of diet, physical activity level, sleep quality and quantity, stress, and medication use, among others, may vary throughout the lifecycle and substantially impact disease pathophysiology.^8^ Clearly, differences in exposure to environmental factors that influence body weight control and glucose homeostasis, together with a unique genetic background in humans, contribute to interindividual differences in the pathophysiology of cardiometabolic diseases. For instance, we have recently demonstrated that there may be distinct tissue-specific insulin resistance phenotypes, characterized by different adipose tissue transcriptome, metabolome as well as lipidome profiles,^9^ representing different etiologies toward Type 2 diabetes and cardiometabolic disease. Clearly, this may also relate to interindividual differences in responses to interventions. Third, no obvious sex-specific effects were found in this study by Petrick et al.^6^ Although the biological underpinnings of sexual dimorphism in metabolic homeostasis become more clear,^10^ deciphering sex differences in adipose tissue function, skeletal muscle, and liver metabolism, and gut microbiota functionality in the etiology of obesity-related insulin resistance and impaired glucose tolerance in humans is a research direction that certainly warrants further attention. Thus, long-term prospective studies in humans are urgently needed to elucidate whether the important mechanistic insight provided by the work from Petrick et al.^6^ in mice translate to humans. This knowledge is essential to develop novel treatment avenues and to optimize current strategies to prevent and treat obesity-related cardiometabolic complications using a more targeted approach for different subgroups of the population.

## Conflict of Interest

The authors have no potential conflicts of interest related to this article.
